# Microscopic and submicroscopic *Plasmodium* infections in indigenous and non-indigenous communities in Colombia

**DOI:** 10.1186/s12936-020-03226-4

**Published:** 2020-04-16

**Authors:** Jehidys Montiel, Lina M. Zuluaga, Daniel C. Aguirre, Cesar Segura, Alberto Tobon-Castaño, Ana M. Vásquez

**Affiliations:** 1 Grupo Malaria-Facultad de Medicina, Universidad de Antioquia, Carrera 53 No. 61–30, Lab 610, Medellín, Colombia; 2 Instituto de Investigaciones Médicas, Facultad de Medicina, Universidad de Antioquia, Medellín, Colombia

**Keywords:** Malaria, Indigenous communities, Asymptomatic infections, Submicroscopic infections

## Abstract

**Background:**

The indigenous population is considered a highly susceptible group to malaria because individuals usually live in areas with high exposure to *Anopheles* and poverty, and have limited access to health services. There is a great diversity of indigenous communities in Colombia living in malaria-endemic areas; however, the burden of infection in these populations has not been studied extensively. This study aimed to determine the prevalence of *Plasmodium* infections in indigenous and non-indigenous communities in two malaria-endemic areas in Colombia.

**Methods:**

A community-based cross-sectional survey was conducted in seven villages of Turbo and El Bagre municipalities; three of these villages were indigenous communities. Inhabitants of all ages willing to participate were included. Sociodemographic and clinical data were recorded as well as household information. The parasitological diagnosis was performed by microscopy and nested PCR. The prevalence of microscopy and submicroscopic infection was estimated. An adjusted GEE model was used to explore risk factors associated with the infection.

**Results:**

Among 713 participants, 60.7% were from indigenous communities. *Plasmodium* spp. was detected in 30 subjects (4.2%, CI 95% 2.9–5.9); from those, 29 were in the indigenous population, 47% of infections were afebrile, and most of them submicroscopic (10/14). Microscopic and submicroscopic prevalence was 2.5% (CI 95% 1.6–3.9) and 1.7% (CI 95% 0.9–2.9), respectively. In El Bagre, all infections occurred in indigenous participants (3.9%, CI 95% 2.2–7.1), and 81% were submicroscopic. By contrast, in Turbo, the highest prevalence occurred in indigenous people (11.5%; CI 95%: 7.3-17.5), but 88.8% were microscopic. Living in an indigenous population increased the prevalence of infection compared with a non-indigenous population (PR 19.4; CI 95% 2.3–166.7).

**Conclusion:**

There is a high proportion of *Plasmodium* infection in indigenous communities. A substantial proportion of asymptomatic and submicroscopic carriers were detected. The identification of these infections, not only in indigenous but also in the non-indigenous population, as well as their associated factors, could help to implement specific malaria strategies for each context.

## Background

Although significant advances have been made towards malaria elimination in several endemic countries, malaria remains a significant public health problem [1]; the World Malaria Report 2018 estimated that the global malaria burden was around 219 million reported cases and 435,000 deaths worldwide [2]. Besides, the situation in the Americas presents further challenges for control and malaria elimination, given the high proportion of cases of *Plasmodium vivax* infection [3]. Particularly in Colombia, the number of malaria cases officially reported in 2018 was 63,143; with Chocó, Nariño, Cordoba, and Antioquia, the departments with the highest number of malaria cases (27.0%, 20.6%, 15.6%, and 8.8%, respectively) [4].

In the Americas, the indigenous population is considered one of the most vulnerable groups to suffer from malaria. The elevated vulnerability is not solely explained by the fact that individuals live in areas with a high *Anopheles* bite exposure, but also because they have high poverty rates and little to no access to diagnostic and treatment services [5]. Information about health conditions of these populations is not always collected, so their risk is not well understood, but in general, it is known that indigenous communities have poor health indicators as compared to non-indigenous populations, including the morbidity and mortality due to transmissible diseases, child undernutrition, infant mortality rates, and years of potential life lost [6].

In Colombia, 3.4% of the population is indigenous, and there are around 710 indigenous communities located in 27 departments [7], many of them living in malaria-endemic regions. Between 2009 and 2014, 75.8% of the Colombian indigenous population was at risk of being infected by any microorganism, where *Plasmodium* spp. caused 46.7% of the total infections [8]. Unfortunately, there are few studies in the indigenous population, so that the risk is not well understood. Only eight of the 21 malaria-endemic countries of the Americas Region reported cases of ethnic groups and indigenous peoples in 2014 [5]. Without adequate data, it is difficult to follow up on malaria trends, recognize the risk factors in these communities, and establish malaria control strategies. In Colombia, the majority of malaria cases occur at the Pacific coast and Amazon region and affects mainly Afro-Colombian and indigenous populations [1, 9]. In 2018, 62,141 malaria cases were reported, of these, 14,714 (23.7%) were in the indigenous population [10]. Autochthonous malaria transmission has also been reported mainly among indigenous communities in Chocó (Pacific coast) [11].

Antioquia was one of the departments with the highest malaria prevalence in Colombia for many years. However, the cases have decreased markedly from 20,511 in 2008 to 4971 in 2017 [12]. This could be explained by the several malaria control strategies implemented between 2007 and 2010, such as vector control activities, strengthening of diagnosis network, distribution of insecticide-treated bed nets (ITNs), and chemoprophylaxis. Despite this reduction, a significant proportion of malaria cases is related to gold-mining activities, which play an important role in the maintenance of malaria transmission and are considered to be an important barrier to malaria elimination [13]. Like miners, indigenous populations are also considered a significant reservoir of malaria transmission. Unfortunately, these populations have been scarcely studied, and there is not enough information about the *Plasmodium* prevalence in them.

One of the main challenges of malaria control programs is the early diagnosis and treatment, not only for symptomatic but also for asymptomatic infections, which represent a silent reservoir of parasites [14]. Compared to patients with acute malaria disease, who generally seek treatment in health facilities, people with low-density infections that often are asymptomatic, do not seek medical attention or anti-malarial treatment [15, 16]. These infections can contribute to local transmission in an endemic region [17]. It has been reported that in Peru, 50% of *Plasmodium falciparum* and 22% of *P. vivax* asymptomatic infections can harbor gametocytes [18]; similarly, in Colombia, 57% of the samples from asymptomatic volunteers were infective to mosquitoes [19].

In Colombia, the prevalence of low-density infections by *Plasmodium* has been previously explored, finding frequencies from 2 to 15% in general population, with most of the infections being submicroscopic [20–22]. In pregnant women frequencies reached from 1.1% in peripheral to a 2.1% in placental blood [23]. In the Urabá region located in Antioquia-Colombia, the prevalence of asymptomatic infections detected by PCR was 2.6% [24]. Together these studies suggest that in low endemic settings such as Colombia, molecular tests are more useful than microscopy to detect this kind of infection [25–27].

Most of the studies about low-density infections in Colombia have been conducted in the general population and pregnant women, but there are not reports in special populations such as indigenous people. This study aimed to determine the prevalence of microscopic and submicroscopic *Plasmodium* infections in indigenous and non-indigenous communities in two malaria-endemic areas in Antioquia-Colombia and to explore the associated factors to the *Plasmodium* infections.

## Methods

### Study design

A cross-sectional study was conducted in seven malaria-endemic villages of Turbo and El Bagre municipalities between November 2016 and June 2017. Villages were selected based on the historical records of malaria cases, and the accessibility for field staff. Three of these villages were indigenous communities (Los Aguacates and Los Almendros in El Bagre and Arqua in Turbo).

### Study sites description

**Fig. 1:**
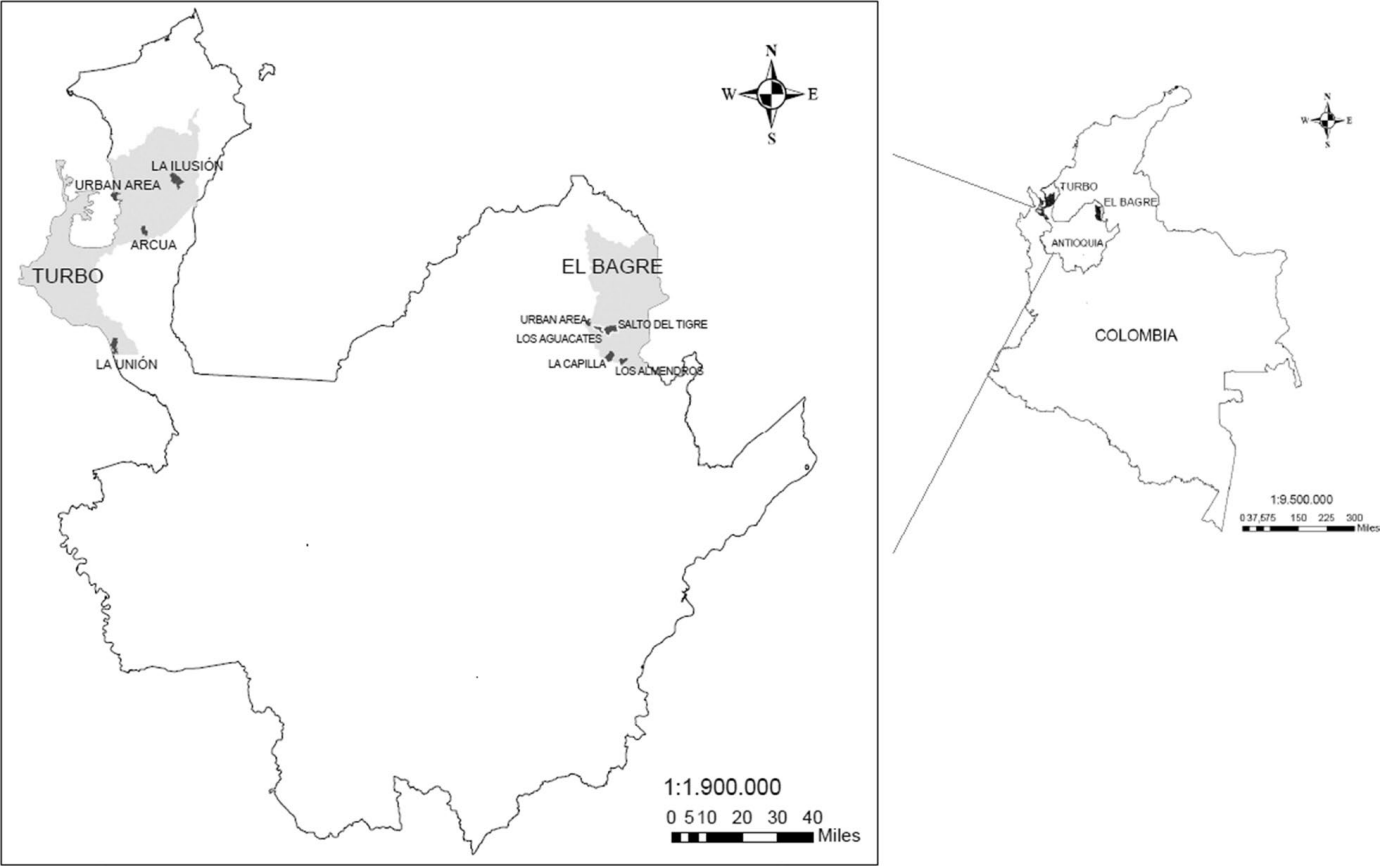
Study sites in Antioquia department, Colombia

The two towns selected for this study are located in the Antioquia department (Fig. 1). Turbo (8° 05′ 42″ N, 76° 44′ 123″) is located in the Urabá region of Colombia, has an area of 3055 m^2^, an altitude of 2 metres above sea level and an average temperature of 28 °C. The main economic activity is banana production [28, 29]. El Bagre (7° 35′ 25″ N, 74° 48′ 27″), is located in the Bajo Cauca region of Colombia, has an area of 1563 m^2^, an average temperature of 32 °C and an altitude of 50 metres above sea level [29, 30]. The main economic activity is gold mining. The annual parasite index (parasite incidence per 1000 population) reported for 2017 was 0.77 in Turbo and 21.29 in El Bagre, in both, *P. vivax* was the predominant species [12].

### Study population and sample collection

A census was carried out in each village to know the number of houses and people; all individuals were invited to participate in the study. Individual and household data regarding sociodemographic, self-report of previous malaria episodes, and characteristics of the household were collected. Axillary temperature was measured, and 6 mL of whole blood was collected by venipuncture in tubes with anticoagulant EDTA (BD Vacutainer, BD Franklin Lakes, USA). Samples were stored at 4 °C until processing at the laboratory of the local hospital in each municipality. The sample was used to perform diagnosis by microscopy using thick and thin blood smears and to measure hemoglobin levels using by a HemoCue (Hb 201+, Lake Forest, California). A blood sample was dropped on to Whatman filter paper #3 (Fisher, Ref 1003-917) and later used for molecular diagnosis. Mild anaemia was defined as a hemoglobin level between 10 g/dL and 10.9 g/dL, moderate anaemia between 7 g/dL and 9.9 g/dL and severe anaemia below 7 g/dL following World Health Organization criteria [2].

### Laboratory procedures

#### Conventional microscopy

Field-stained thick and thin blood smears were read in the field by an expert microscopist according to national guidelines [31] with a limit of detection (LOD) around 50 parasites/μL [32]. Parasitaemia was estimated against 200 leukocytes (assuming a standard value of 8000 leukocytes/μL of blood) and was expressed as parasites/μL (p/μL). A sample was considered negative if, after the examination of 200 microscopic fields at 100× magnification, no parasites were observed. As a quality control, a second reading was performed in all PCR and/or microscopy positives samples and 10% of negative ones. Discordant results were solved by a third reading.

All participants with positive thick smear received anti-malarial treatment according to national guidelines; *P. vivax* cases were treated with chloroquine for 3 days and primaquine (PQ) for 14 days, *P. falciparum* cases were treated with artemether plus lumefantrine for 3 days [31].

#### Molecular diagnostic

DNA was extracted from half a blood spot of the filter cards (approximately 30 µL of blood) using the QIAamp DNA Mini Kit following the manufacturer’s recommendations (QIAGEN, Germany). Nested PCR was performed as a two-step procedure following the protocol described by Singh et al. with a LOD of 1–6 parasites/μL [33]. Amplification products were resolved in a 1.5% agarose gel stained with GelRed™ (Biotium, United States) and visualized under UV light. This protocol consists of a universal PCR that detects and amplifies a region of the 18S ribosomal gene from the *Plasmodium* genus. Positive samples for *Plasmodium* spp. were further processed using species-specific primers for *P. falciparum* and *P. vivax.*

#### Ethical considerations

This study was reviewed and approved by the Ethics Committee of the Medicine school, Universidad de Antioquia, Colombia (Record 011 dated 28 July 2016). Before to start the fieldwork, permission from community leaders was approved in each village. Signed informed consent was obtained before the interview and blood sampling of all participants. In the case of individuals < 18 years old, an additional informed consent from parents or legal guardians was also obtained.

#### Statistical analysis

All data from questionnaires and forms were entered into a Microsoft Access database, and statistical analyses were conducted in STATA 14 (StataCorp. 2015. Stata Statistical Software: Release 14. College Station, TX: StataCorp LP). A description of the population was carried out for both, individual (anaemia, gender, age group, occupation, residence time in endemic region, number of malaria episodes, malaria in the previous year, municipality, use of mosquito nets) and housing factors (inhabitants per household, draining of stagnant water, access to electricity, water and sewer system, mosquito mesh on windows, roof and wall materials). Frequencies were expressed as numbers and percentages for qualitative variables and medians with the interquartile range for the quantitative ones. The prevalence of *Plasmodium* infection was estimated by diagnostic tests (microscopy and nPCR), locality (Turbo and El Bagre), and type of community (indigenous and non-indigenous communities). 95% confidence intervals (CI) are shown for each estimation. A generalized estimating equation (GEE) model [34] was used to handle the nested structure of sampled data (713 individuals nested within 212 households), GEE model was fitted for a Poisson family with a logarithmic link and an exchangeable correlation. Crude (PR) and adjusted (aPR) Prevalence ratio (by occupation and malaria in the previous year) were calculated for the *Plasmodium* infection (outcome) with 95% confidence intervals for each factor.

## Results

### Socio-demographic and household characteristics

**Fig. 2:**
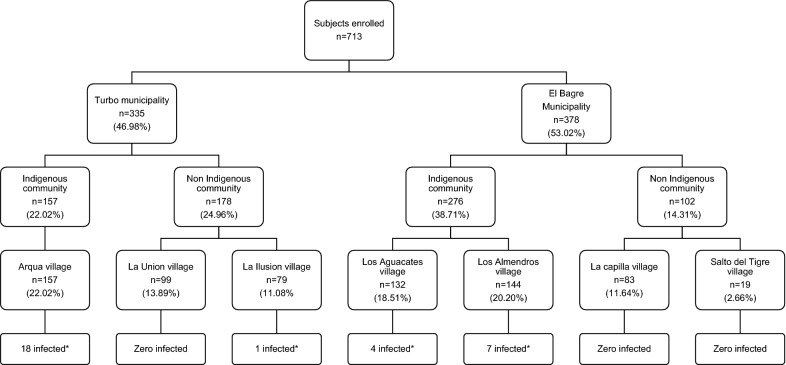
Flowchart of enrolled participants and proportion of infected people by villages. *Plasmodium infections detected by either microscopy or PCR

In this study, 713 subjects from 212 houses were enrolled in seven villages of two municipalities in the Antioquia department. El Bagre town had more indigenous people (n = 276; 38.7%) than Turbo (n = 157; 22.0%). The proportion of *Plasmodium* infection in both municipalities was higher in indigenous communities than non-indigenous communities (6.69% vs. 0.36%) (Fig. 2).

The characteristics of participants, according to the residence in the indigenous communities, are shown in Table 1. There was a slightly higher proportion of anaemia in non-indigenous people (15.4%) compared to indigenous (12.7%). Additionally, the proportion of moderate anaemia was higher in indigenous communities than non-indigenous communities (11.6% and 32.7%, respectively). The same pattern was observed in severe anaemia (2.4% and 3.6% respectively).

**Table 1:** Clinical and demographic characteristics and malaria history in the study population

Characteristic	Non-Indigenous community		Indigenous community		Total	
	n = 280		n = 433		n = 713	
	n	%	n	%	n	%
Individual characteristics						
Haemoglobin < 11 mg/dL	43	15.4	55	12.7	98	13.7
Gender						
Female	145	51.8	234	54.0	379	53.2
Age						
< 5	27	9.6	43	9.9	70	9.8
5–15	87	31.1	133	30.7	220	30.9
> 15	166	59.3	255	58.9	421	59.0
Occupation						
Outdoor	152	54.3	207	47.8	359	50.4
Residence time in endemic region						
≥ 5 years	210	75.0	314	72.5	524	73.5
Self-report of number of symptomatic malaria episodes						
0	106	37.9	173	40.0	279	39.1
1	58	20.7	66	15.2	124	17.4
> 1	116	41.4	194	44.8	310	43.5
Self-report of malaria last year	12	4.3	51	11.8	63	8.8
Municipality						
Turbo	102	36.4	157	36.3	259	36.3
El Bagre	178	63.6	276	63.7	454	63.7
Use of bed net	262	93.6	405	93.5	667	93.5
Household characteristics						
Number of inhabitants per household						
1–4	127	45.4	162	37.4	289	40.5
> 5	146	52.1	256	59.1	402	56.4
Participants who have animals in their households	259	92.5	420	97.0	679	95.2
Participants who drain standing water in their households	78	27.9	20	4.6	98	13.7
Participants who have no access to electricity in their households	23	8.2	149	34.4	172	24.1
Participants who have no access to water in their households	266	95.0	382	88.2	648	90.9
Participants who have no access to sewage system in their households	271	96.8	415	95.8	686	96.2

The gender and age distribution was similar in both groups, and the majority of people have lived in an endemic malaria region for more than four years (72.5% and 75% in non-indigenous and indigenous communities, respectively). Most of the indigenous people lived in houses with more than five people (59.1%) and had animals in their homes (97%). Additionally, a high proportion of them did not drain standing water in their homes (93%), and 34% did not have access to electricity.

### *Plasmodium* infections prevalence

**Fig. 3:**
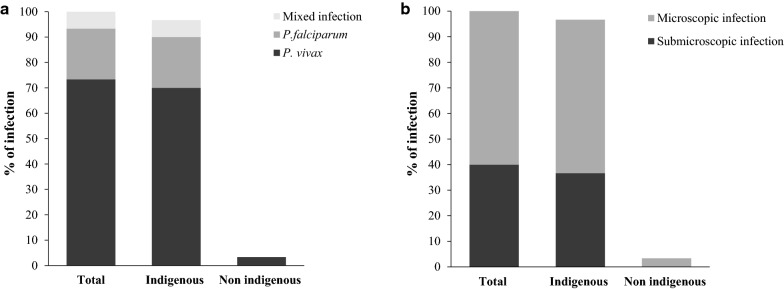
Proportion of *Plasmodium* infection in indigenous and non-indigenous communities. **a** Proportion of *Plasmodium* infection by species. **b** Proportion of *Plasmodium* infection by diagnostic test (Microscopic infection were detected by microscopy and PCR and submicroscopic infection were only detected by PCR)

The overall prevalence of *Plasmodium* spp. infection was 4.21% (95% CI 2.95–5.96%), 60% of these infections were detected by microscopy, and *P. vivax* was the predominant species (3.09%; 95% CI 2.04–4.65%). The prevalence of infection was higher in Turbo compared to El Bagre (5.67%, 95% CI 3.64–8.73% versus 2.91%, 95% CI 1.62–5.19% respectively); most of the infections in Turbo were detected by microscopy (16/19, 84.2%) and 93.75% (15/16) had malaria symptoms (Table 2); while most of the infections in El Bagre were asymptomatic (temperature < 37.5 °C) and submicroscopic (9/11). In both towns, a considerably higher prevalence of *Plasmodium* was found in indigenous communities (Fig. 3), resulting in a 12-fold increase in the prevalence of malaria as compared to non-indigenous communities.

**Table 2:** Overall *Plasmodium* prevalence in the study population by diagnostic test

Municipality	Overall prevalence					Prevalence detected by microscopy and PCR					Prevalence detected only by PCR				
	n	%	95% CI			n	%	95% CI			n	%	95% CI		
Total n = 713	30	4.21	2.95	–	5.96	18	2.52	1.59	–	3.98	12	1.68	0.96	–	2.94
*P. falciparum*	6	0.84	0.38	–	1.86	3^a^	0.42	0.14	–	1.30	3	0.42	0.14	–	1.30
*P. vivax*	22	3.09	2.04	–	4.65	15^b^	2.10	1.27	–	3.46	7	0.98	0.47	–	2.05
Mixed infection	2	0.28	0.07	–	1.12	0	0.00	0.00	–	0.00	2	0.28	0.07	–	1.12
El Bagre n = 378	11	2.91	1.62	–	5.19	2	0.53	0.13	–	2.10	9	2.38	1.24	–	4.52
Indigenous community n = 276	11	3.99	2.22	–	7.07	2	0.72	0.18	–	2.86	9	3.26	1.70	–	6.16
Non-indigenous community n = 102	0	0.00	0.00	–	0.00	0	0.00	0.00	–	0.00	0	0.00	0.00	–	0.00
Turbo n = 335	19	5.67	3.64	–	8.73	16	4.78	2.94	–	7.66	3	0.90	0.29	–	2.75
Indigenous community n = 157	18	11.46	7.33	–	17.50	16	10.19	6.32	–	16.02	2	1.27	0.32	–	4.98
Non-indigenous community n = 178	1	0.56	0.08	–	3.91	0	0.00	0.00	–	0.00	1	0.56	0.08	–	3.91

^a^Median parasitaemia (IQR: inter quartile range) [parasites/µl]) 360 (40–28,186)

^b^Median parasitaemia (IQR) [parasites/µl]) 3147 (2805–15,615)

### Association between *Plasmodium* infections and risk factors

The analysis of the association of the subjects and household characteristics with *Plasmodium* infections is shown in Table 3. The prevalence of *Plasmodium* infections was higher in males (5.7%) than females (2.9%), with an aPR for the male sex of 2.38 (95% CI 1.07–5.31). People who have lived in the endemic region for more than 4 years had a higher prevalence of infection than those living in the endemic region for less time (4.2% and 2.3%, respectively). The infection was also higher in people with previous self-reported malaria as compared to people with no malaria history (4.8% and 3.2%, respectively). Furthermore, living in an indigenous community increased the prevalence e of infection as compared to the non-indigenous population (aPR 17.86, 95% CI 2.12–150.19). Regarding household factors, it was found that participants with no electricity services in their households have a higher prevalence of *Plasmodium* infection (aPR 4.62, 95% CI 1.89–11.32). The PR could not be calculated for the association with water and sewage services (the convergence was not achieved in the GEE model); because none of the infected people had access to these services.

**Table 3:** Association between individual and household characteristics with *Plasmodium* infections

Characteristic	Infected by *Plasmodium*				Crude PR^a^				Adjusted PR^b^			
	No		Yes		PR	IC 95%			PR	IC 95%		
	n	%	n	%								
Individual factors												
Sex												
Female	368	97.1	11	2.9	1				1			
Male	315	94.3	19	5.7	1.95	0.95	–	4.02	2.38	1.07	–	5.31
Age												
Median (IQR)	20	(10–37)	17	(11–29)	0.99	0.97	–	1.01	0.99	0.97	–	1.01
Age												
< 5	67	95.7	3	4.3	1				1			
5–15	209	95.0	11	5.0	1.29	0.36	–	4.61	0.93	0.20	–	4.48
> 15	405	96.2	16	3.8	0.94	0.27	–	3.22	0.68	0.15	–	3.07
Residence time in endemic region												
Median (IQR)	10	(4–20)	12	(6–20)	1.00	0.97	–	1.03	1	0.97	–	1.03
Residence time in endemic region												
< 5 years	172	97.7	4	2.3	1				1			
≥ 5 years	502	95.8	22	4.2	1.64	0.57	–	4.70	1.83	0.55	–	6.08
Number of episodes of symptomatic malaria												
Median (IQR)	1	(0–3)	2	(0–4)	1.02	0.99	–	1.06	1.01	0.98	–	1.05
0	270	96.8	9	3.2	1				1			
1	120	96.8	4	3.2	0.98	0.30	–	3.21	0.75	0.19	–	2.92
> 1	293	94.5	17	5.5	1.77	0.78	–	4.03	1.55	0.59	–	4.10
Self-report of malaria history												
0	270	96.8	9	3.2	1				1			
≥ 1	413	95.2	21	4.8	1.53	0.70	–	3.36	1.26	0.50	–	3.19
Participants living in the indigenous community												
No	279	99.6	1	0.4	1				1			
Yes	404	93.3	29	6.7	20.77	2.14	–	201.12	17.86	2.12	–	150.19
Use of bed net												
Yes	638	95.7	29	4.3	1				1			
No	42	97.7	1	2.3	0.44	0.05	–	4.10	0.51	0.06	–	4.14
Household factors												
Number of inhabitants per household												
Median (IQR)	5	(4–6)	4	(4–7)	1.08	0.90	–	1.31	1.05	0.88	–	1.27
1–4	280	96.9	9	3.1	1				1			
≥ 5	381	94.8	21	5.2	1.47	0.62	–	3.44	1.38	0.59	–	3.20
Participants who have animals in their households												
No	24	96.0	1	4.0	1				1			
Yes	650	95.7	29	4.3	0.88	0.12		6.51	0.79	0.10	–	5.97
Participants who drain standing water in their households												
Yes	97	99.0	1	1.0	1				1			
No	577	95.2	29	4.8	3.99	0.48	–	32.88	3.90	0.49		30.78
Participants who have access to electricity in their households												
Yes	518	97.7	12	2.3	1.00				1			
No	154	89.5	18	10.5	4.94	2.17		11.28	4.62	1.89	–	11.32
Participants who have access to water in their households												
Yes	56	100.0	0	0.0	1				1			
No	618	95.4	30	4.6	CNA^c^				CNA			
Participants who have access to sewage system in their households												
Yes	18	100.0	0	0.0	1				1			
No	656	95.6	30	4.4	CNA				CNA			

^a^Prevalence Ratio, ^b^ Adjusted by occupation and malaria last year, ^c^ Convergence not achieved

Living in an indigenous community as well as some characteristics of the houses were associated with a higher incidence rate of *Plasmodium* infections. A household description of indigenous and non-indigenous communities is detailed in Table 4. A total of 125 from 212 houses belong to indigenous communities (59%), 30.4% of these did not have electricity service, and 91.2% did not drain the standing water; these proportions were substantially higher in indigenous compared to non-indigenous communities.

**Table 4:** Household characteristics by indigenous and non-indigenous communities

Variable	Non-indigenous community		Indigenous community		Total	
	n = 87		n = 125		n = 212	
	n	%	n	%	n	%
Number of inhabitants						
1–4	55	63.2	70	56.0	125	59.0
> 5	31	35.6	51	40.8	82	38.7
Ownership						
Own	79	90.8	113	90.4	192	90.6
Leased	8	9.2	9	7.2	17	8.0
Electric service						
Yes	77	88.5	84	67.2	161	75.9
No	9	10.3	38	30.4	47	22.2
Water service						
Yes	5	5.7	12	9.6	17	8.0
No	82	94.3	110	88.0	192	90.6
Sewage system						
Yes	3	3.4	4	3.2	7	3.3
No	84	96.6	118	94.4	202	95.3
Kind of water bodies						
Lake	3	3.4	2	1.6	5	2.4
Stagnant rain water	6	6.9	3	2.4	9	4.2
River	77	88.5	114	91.2	191	90.1
Fumigation (vector control programme)						
Yes	78	89.7	115	92.0	193	91.0
No	9	10.3	7	5.6	16	7.5
Use of insecticides						
Yes	5	5.7	10	8.0	15	7.1
No	82	94.3	112	89.6	194	91.5
Draining standing water						
Yes	23	26.4	8	6.4	31	14.6
No	64	73.6	114	91.2	178	84.0
Mosquito mesh for windows						
Yes	0	0.0	7	5.6	7	3.3
No	87	100.0	115	92.2	202	95.3
Wall material						
Wood	74	85.1	103	82.4	177	83.5
Cement blocks	10	11.5	10	8.0	20	9.4
Others	3	3.4	9	7.2	12	5.7
Roof material						
Palm leaf	29	33.3	19	15.2	48	22.6
Mud	7	8.0	13	10.4	20	9.4
Tin	49	56.3	90	72.0	139	65.6
Plastic	2	2.3	0	0.0	2	0.9

## Discussion

This study evaluated the prevalence of microscopic and submicroscopic *Plasmodium* infections in indigenous and non-indigenous communities from Antioquia, Colombia, and its associated factors, to describe the distribution of disease prevalence among heterogeneous populations; this knowledge is necessary to implement proper control strategies for each context [35]. We found that the prevalence of *Plasmodium* infections it was increased 12-fold in indigenous communities as compared to non-indigenous communities in both municipalities. Even more, all infections in El Bagre were detected in indigenous communities (11/11), and most of them were asymptomatic and submicroscopic (9/11). On the contrary, most of the infections in indigenous communities in Turbo were symptomatic and microscopic (84.2%).

It is known that malaria transmission in Colombia varies among the endemic regions [35]; in this way, these findings could be explained by differences in malaria profiles in each municipality. Although the general prevalence of malaria in Antioquia has decreased in recent years, the number of cases in El Bagre has been higher than in Turbo (from 190.45 cases/1000 people in 2007 to 21.29 cases/1000 people in 2017 in El Bagre and from 61.53 cases/1000 in 2007 to 0.77 cases/1000 people in 2017 in Turbo).

Most of the malaria cases were caused by *P. vivax* and it is well known that the PQ used for the *P. vivax* treatment can induce haemolytic crises in individuals with glucose 6-phosphate dehydrogenase (G6PD) deficiency. The G6PD deficiency is distributed worldwide, however, its frequency varies among regions and ethnic groups. A previous report in malaria-endemic areas of Colombia located on the Pacific coast, found a frequency of G6PD deficiency of 6.56% [36], studies in indigenous populations (Amerindian) are lacking. Considering the high number of *P. vivax* infections found in these populations, there is a need for further evaluation of the frequency of G6PD deficiency in malaria-endemic areas in view that the primaquine treatment (14 days) is required for the radical cure.

As previously reported, malaria immunity is determined by previous *Plasmodium* exposure, where an anti-disease immunity is first achieved, resulting in a reduction of severe malaria and mortality. Then, an anti-parasitic immunity is slowly acquired and confers protection against high parasitic densities, which in turn protect against the severe disease [37]; this could explain the highest prevalence of submicroscopic infections in El Bagre, where 50.3% of individuals had had more than one malaria episode over life compared to 35.8% in Turbo (Additional file 1: Table S1). Nevertheless, it was not found an association of this variable with the *Plasmodium* infections using a GEE analysis. However, a previous study in Nariño- Colombia showed that having suffered from more than one malaria episode was associated with an increased risk of having asymptomatic infections (aOR 2.4, 95% CI 1.1–5.4) [22]. These differences could be explained because this model included not only asymptomatic but also symptomatic infections.

Household factors are also associated with malaria risk [38]. It was observed that having no access to electricity was associated with an increase in the malaria rate. These findings are in agreement with previous studies that reported that the poorest households had a 29% greater risk of microscopic parasitaemia compared to the poorest houses (aRR 1.29; 95% CI 1.07–1.55) [39]. Additionally, lack of household electricity increased the childhood mortality in Rwanda, including malaria mortality (aOR: 1.4, 95% CI 1.0–1.8) [40]. The above is important because housing quality can affect malaria risk through its effect on house entry of the malaria vector [39].

Taken together, the individual and housing characteristics could help to understand why the indigenous population has a higher prevalence than its counterpart, the non-indigenous population does. Ethnicity is an important determinant of health conditions, influencing the morbidity and mortality rates in different ethnic groups and interfering with access to health services for some populations [41]. In Colombia, the exclusion of indigenous people is reflected in poverty rates, lack of land and employment, school desertion, unsatisfied basic needs, a higher prevalence of transmissible diseases and limited access to health services compared to the general Colombian population [42]. Regarding this last point, it was found that the indigenous villages were farther from the health services (1 to 2 h by motorcycle) compared to the non-indigenous villages, and the road conditions were far worse. Furthermore, the indigenous population frequently lives close to rainforests or wetlands where they have more vector exposure, resulting in an increased risk of getting sick with vector-borne diseases such as malaria [8].

It is possible to suggest that the diversity of epidemiologic characteristics of malarial infection among the Colombian subpopulations account for an ideal environment for parasite evolution. In this environment, the parasite can interact with susceptible populations from different ethnicities and under different public health interventions [35]. The prevention efforts should be population-specific and vary according to the individual, housing, and environmental characteristics. Given the heterogeneity of the prevalence of malaria in Colombia, it has become necessary to adjust malaria control activities according to each population and context.

Further studies are needed to evaluate the potential integration of molecular tests into the surveillance programs to promptly detect malaria infection in the community in order to contribute to the control and future elimination strategies. However, due the low prevalence of infection in this region of Colombia, there is also a need to evaluate the costs per assay comparing to conventional test, including equipment, reagents, staff, training, and maintenance in order to evaluate the cost-effectiveness of molecular test for their potential integration into surveillance strategies and explore alternatives as serological surveillance. A previous study in a low transmission setting in Indonesia, suggested that reactive-active detection of cases in the community using molecular test had high costs per individual screened, however, compared to microscopy, molecular test was most cost-effective for the detection of infections [43]. Also, another study in a low transmission setting in Africa concludes that standards test, such as rapid diagnosis test (RDTs), are not useful to detect infections in the community and suggest that the achievement of malaria elimination may require active case detection using more sensitive point-of-care diagnostics, especially in high-risk groups [44], that in our cases are the indigenous communities among others.

This study has some limitations. First, due to cross-sectional design, the association with malaria status should be interpreted with caution, as they do not imply causality. Second, it was no possible to analyse the risk factors for asymptomatic infections exclusively due to the low number of this type of infection. This last could, in turn, affect the accuracy of confidence intervals for some of the factors analysed due to the sample size. Third, as mentioned before, the villages in this study were selected based on the historical records of malaria cases, the distance to the urban area, and the accessibility for field staff, and a random selection of the villages included in the study was not performed. The results of this study cannot be extrapolated to the general population; nevertheless, they are useful to exhibit the problems around the asymptomatic infections in the indigenous and non-indigenous people. Fourth, considering that nowadays there are ultra-sensitive molecular tests for the detection of low-density infections, the prevalence in this study could be underestimated due to the limit of detection of the nPCR used, nevertheless, the nPCR used in this study was able to detect 1.6 times more infections than microscopy showing the presence of a significant *Plasmodium* reservoir in the region. At last, it is possible that other variables which were not considered in the GEE model could explain the associated factors to *Plasmodium* infections. Future studies are required to improve the knowledge about the risk factors of the *Plasmodium* infections in indigenous communities. Despite these limitations, these results are useful to understand malaria transmission in studied places and to suggest prevention efforts according to the individual, housing, and environmental characteristics.

## Conclusion

This study reveals that in both municipalities, most of the *Plasmodium* infections were in indigenous communities. Nevertheless, the infection profile was different for each town. A substantial proportion of asymptomatic and submicroscopic carriers were detected in El Bagre, while most of the symptomatic and microscopic infections were identified in Turbo. These findings provide an understanding of the key characteristics of asymptomatic, submicroscopic, and microscopic infections in the study population: to live in an indigenous community, having had previous malaria episodes and not having access to electricity, sewage system, and water services. The current malaria control efforts could benefit from the implementation of targeted interventions in indigenous villages using molecular tests to identify submicroscopic reservoirs that could be contributing to malaria transmission, although further studies are needed to evaluate the potential integration of molecular test into the surveillance programs to promptly detect malaria infection in the community, especially in high-risk groups. Additionally, the identification of these infections not only in indigenous but also in the non-indigenous populations, as well as demographic, social and household factors related to them, could help to implement specific malaria strategies for each context.

## Supplementary information

**Additional file 1.** Additional table.

## Data Availability

The datasets used and/or analysed during the current study are available from the corresponding author on reasonable request.

## Abbreviations

aPR: Adjusted prevalence ratio
aOR: Adjusted odds ratio
CI: Confidence interval
DNA: Deoxyribonucleic acid
GEE: Generalized estimating equation
G6PD: Glucose 6-phosphate dehydrogenase
PR: Prevalence ratio
IQR: Inter quartile range
ITNs: Insecticide-treated bed nets
LOD: Limit of detection
p/μL: Parasite/μL
PCR: Polymerase chain reaction
nPCR: Nested polymerase chain reaction
PQ: Primaquine
spp: Species
UV: Ultraviolet
