# Supplementary material for: Microscopic and submicroscopic Plasmodium infections in indigenous and non-indigenous communities in Colombia
Source: Malar J. 2020 Apr 16;19:157. doi: 10.1186/s12936-020-03226-4 (PMC7164158; doi:10.1186/s12936-020-03226-4)
Supplement: Supplementary file 1 — Additional file 1. Additional table. [file 12936_2020_3226_MOESM1_ESM.docx]

**Additional Table S1**. Clinical and demographic characteristics and malaria history in the study population by site.

| **Characteristic** |  | **El Bagre** | | |  | **Turbo** | | |  | **Total** | | |
| --- | --- | --- | --- | --- | --- | --- | --- | --- | --- | --- | --- | --- |
|  |  | **n= 378** | | |  | **n= 335** | | |  | **n=713** | | |
|  |  | **n** |  | **%** |  | **n** |  | **%** |  | **n** |  | **%** |
| Hemoglobin <11g/dL |  | 35 |  | 9.3 |  | 63 |  | 18.8 |  | 98 |  | 13.7 |
| **Sex** |  |  |  |  |  |  |  |  |  |  |  |  |
| Female |  | 202 |  | 53.4 |  | 177 |  | 52.8 |  | 379 |  | 53.2 |
| **Age** |  |  |  |  |  |  |  |  |  |  |  |  |
| <5 |  | 31 |  | 8.2 |  | 39 |  | 11.6 |  | 70 |  | 9.8 |
| 5 -15 |  | 121 |  | 32.0 |  | 99 |  | 29.6 |  | 220 |  | 30.9 |
| > 15 |  | 226 |  | 59.8 |  | 195 |  | 58.2 |  | 421 |  | 59.0 |
| **Occupation** |  |  |  |  |  |  |  |  |  |  |  |  |
| Outdoor |  | 223 |  | 59.0 |  | 136 |  | 40.6 |  | 359 |  | 50.4 |
| **Residence time in endemic region** |  |  |  |  |  |  |  |  |  |  |  |  |
| ≥5 years |  | 287 |  | 75.9 |  | 237 |  | 70.7 |  | 524 |  | 73.5 |
| **Number of episodes of symptomatic malaria** |  |  |  |  |  |  |  |  |  |  |  |  |
| 0 |  | 128 |  | 33.9 |  | 151 |  | 45.1 |  | 279 |  | 39.1 |
| 1 |  | 60 |  | 15.9 |  | 64 |  | 19.1 |  | 124 |  | 17.4 |
| >1 |  | 190 |  | 50.3 |  | 120 |  | 35.8 |  | 310 |  | 43.5 |
| **Self-report of malaria last year** |  |  |  |  |  |  |  |  |  |  |  |  |
| Yes |  | 23 |  | 6.1 |  | 40 |  | 11.9 |  | 63 |  | 8.8 |
| **Participants living in the indigenous community** |  | 276 |  | 73.0 |  | 157 |  | 46.9 |  | 433 |  | 60.7 |
| **Use of bed net** |  | 344 |  | 91.0 |  | 323 |  | 96.4 |  | 667 |  | 93.5 |
| **Number of inhabitants per household** |  |  |  |  |  |  |  |  |  |  |  |  |
| 1 - 4 |  | 139 |  | 36.8 |  | 150 |  | 44.8 |  | 289 |  | 40.5 |
| >5 |  | 223 |  | 59.0 |  | 179 |  | 53.4 |  | 402 |  | 56.4 |
| **Participants who have animals in their households** |  | 361 |  | 95.5 |  | 318 |  | 94.9 |  | 679 |  | 95.2 |
| **Participants who use mosquito mesh for windows in their households** |  | 6 |  | 1.6 |  | 24 |  | 7.2 |  | 30 |  | 4.2 |
| Does not apply |  | 3 |  | 0.8 |  | 0 |  | 0 |  | 3 |  | 0.4 |
| **Participants who draining standing water in their households** |  | 98 |  | 25.9 |  | 0 |  | 0.0 |  | 98 |  | 13.7 |
| **Participants who have no access to electricity in their households** |  | 16 |  | 4.2 |  | 156 |  | 46.6 |  | 172 |  | 24.1 |
| **Participants who have no access to water in their households** |  | 313 |  | 82.8 |  | 335 |  | 100 |  | 648 |  | 90.9 |
| **Participants who have no access to sewage system in their households** |  | 351 |  | 92.9 |  | 335 |  | 100 |  | 686 |  | 96.2 |
